# Machine‐Learning Framework for Designing Stable Interfaces in All‐Solid‐State Lithium‐Ion Batteries

**DOI:** 10.1002/advs.76305

**Published:** 2026-06-26

**Authors:** Sehyeok Park, Myeongcho Jang, Hun‐Gi Jung, Kyung Yoon Chung, Seungho Yu

**Affiliations:** ^1^ Energy Storage Research Center Korea Institute of Science and Technology Seongbuk‐gu Seoul Republic of Korea; ^2^ School of Mechanical Engineering Korea University Seongbuk‐gu Seoul Republic of Korea; ^3^ Division of Energy & Environment Technology KIST School Korea University of Science and Technology Seoul Republic of Korea; ^4^ Department of Energy Science and KIST‐SKKU Carbon‐Neutral Research Center Sungkyunkwan University Suwon Republic of Korea

**Keywords:** all‐solid‐state lithium‐ion batteries, coating materials, interfacial stability, ML‐guided screening, supervised learning, unsupervised learning

## Abstract

All‐solid‐state lithium‐ion batteries are promising next‐generation energy‐storage systems, but interfacial instability between cathodes and solid electrolytes remains a major barrier to long‐term durability. Interfacial coatings can mitigate these reactions, yet coating selection is limited by the vast chemical design space and incomplete database coverage. In this study, coating discovery is formulated as a prediction‐and‐design problem in which interfacial reaction energies define a composition‐to‐reactivity map that generalizes to unseen compounds. Reaction energies are calculated for 809 phase‐stable Li‐containing compounds from the Materials Project against 10 oxide cathodes and 7 sulfide solid electrolytes. Unsupervised clustering identifies distinct reactivity groups, and composition‐based analysis reveals signatures, including polyanion tolerance and fixed‐valence cations, that define a low‐reactivity design envelope. Using composition‐derived physicochemical descriptors, an ensemble regressor predicts pair‐averaged reaction energies for newly enumerated compositions. Within this envelope, charge‐balanced Li─M─O and Li─M─A─O compositions (A = B, P, Si) are enumerated, pre‐screened, and validated by phase‐diagram analysis. This workflow enables interpretable machine‐learning‐guided expansion beyond existing databases for scalable coating discovery.

## Graphical abstract

A data‐driven strategy is developed to discover coating materials for all‐solid‐state lithium batteries. Using calculations of interfacial reactivity, unsupervised pattern recognition, and machine‐learning prediction, the study identifies low‐reactivity compositional patterns and screens new lithium‐based oxide and polyanion candidates, extending coating discovery beyond the limited coverage of existing materials databases.

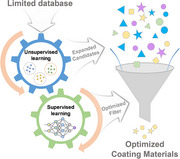

## 1 Introduction

All‐solid‐state lithium‐ion batteries (ASSLIBs) are actively pursued as next‐generation energy‐storage systems because solid electrolytes can improve intrinsic safety and enable new cell architectures [1, 2, 3, 4, 5]. However, solid‐solid contacts introduce chemically and mechanically coupled interfacial challenges [6, 7, 8]. In sulfide‐based ASSLIBs paired with high‐voltage oxide cathodes, the limited oxidative stability of sulfide electrolytes and thermodynamic incompatibility can trigger interfacial decomposition and interdiffusion [9, 10]. The resulting cathode‐electrolyte interphase is often resistive, degrading Li‐ion transport and leading to rapid impedance growth [11, 12]. A coating layer between cathode and solid electrolyte can mitigate these effects by acting as a chemical buffer [13, 14, 15]. A successful coating should satisfy several constraints simultaneously: chemical compatibility with both contacting phases, sufficient Li‐ion transport, and practical formation as a conformal thin layer [16, 17, 18, 19, 20].

To evaluate the chemical compatibility required for effective interfacial coatings, high‐throughput thermodynamic screening based on materials databases provides a scalable approach [13, 21, 22, 23, 24]. Phase‐equilibria–based reaction analysis enables rapid estimation of thermodynamic driving forces across many chemical pairings and suggests equilibrium products expected from bulk thermodynamics [25, 26, 27, 28, 29]. However, database‐driven screening is inherently constrained by a finite set of computed and curated entries, and direct lookup alone does not provide a mechanism to systematically explore continuous composition spaces [30, 31, 32]. This limitation is particularly relevant for coatings, where viable candidates can emerge from multicomponent and compositionally flexible chemistries that are only sparsely represented in existing databases. These considerations motivate strategies that expand the candidate set in a chemically controlled manner while remaining anchored to thermodynamic reference information derived from phase equilibria.

In parallel, data‐driven modeling has emerged as a practical way to cope with sparse and expensive labels in materials discovery: surrogate models trained on limited reference data can rapidly prioritize candidates and guide exploration beyond what can be exhaustively computed or measured [33, 34]. Recent AI and machine‐learning studies have accelerated electrolyte innovation across molecular electrolyte design, solvent optimization, and solid‐electrolyte screening [35, 36, 37]. More broadly, AI‐enabled inorganic materials discovery has increasingly moved toward controlled candidate generation and expansion under physically and chemically meaningful constraints [34, 38, 39]. These advances illustrate how data‐driven approaches can connect sparse reference data with chemically guided candidate exploration. Building on this broader direction, we develop a machine‐learning (ML)‐guided framework for coating‐mediated oxide cathode–sulfide electrolyte interfaces, using database thermodynamics as consistent labels while retaining interpretability and chemical plausibility for interface‐relevant composition exploration.

Building on this paradigm, we label 809 phase‐stable Li‐containing Materials Project compounds using a phase‐diagram‐based interfacial reaction energy evaluated against a representative set of 10 oxide cathodes (OCs) and 7 sulfide solid electrolytes (SSEs), establishing a baseline reactivity landscape. We then perform unsupervised clustering of reaction‐energy profiles to separate candidates into distinct reactivity groups and interpret these groups in composition space to extract compositional signatures that define a chemically controlled design envelope. Using composition‐derived physicochemical descriptors, we train a supervised ensemble model to rapidly predict pair‐averaged reaction energies for charge‐balanced Li─M─O and Li─M─A─O compositions (M: metals; A: anion‐forming elements such as B, P, Si) enumerated within this envelope. Finally, prioritized candidates are subjected to precursor‐based phase‐equilibria checks to ensure thermodynamic consistency, thereby enabling ML‐guided candidate‐set expansion and guided exploration rather than novelty‐driven generation. Overall, this ML‐based framework turns database‐limited thermodynamic screening into an interpretable virtual exploration strategy for prioritizing coating chemistries that stabilize OC–SSE interfaces in ASSLIBs.

## 2 Results

### 2.1 Thermodynamic Reactivity Across OC–SSE and Coating‐Mediated Interfaces

**FIGURE 1:**
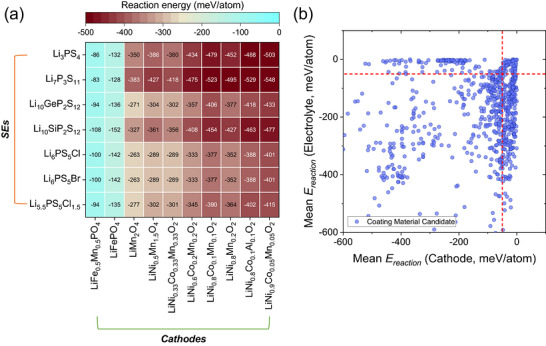
Assessment of interfacial chemical stability. (a) Heatmap illustrating the calculated mutual reaction energies between 7 sulfide SEs and 10 oxide cathode materials. (b) Mean mutual reaction energies of coating material candidates against the 7 sulfide SEs and 10 oxide cathodes. The red dashed line denotes the stability threshold of −50 meV/atom.

The most negative interfacial reaction energies *E_reaction_* between ten oxide cathodes and seven SSEs are summarized as a heatmap in Figure 1a, providing a comparative map of thermodynamic driving forces for interfacial decomposition across practical OC–SSE pairings (more negative values indicate stronger driving forces). Two clearly separated regimes emerge. First, polyanionic olivine cathodes (LiFePO_4_ and LiFe_0.5_Mn_0.5_PO_4_) exhibit consistently weaker driving forces, with *E_reaction_* clustered near −80 to −150 meV/atom across all sulfide chemistries, reflecting their comparatively stable polyanion frameworks. In contrast, oxide cathodes (spinel and layered $R\bar{3}m$ oxides) show substantially more negative *E_reaction_*, typically in the −260 to −550 meV/atom range, indicating strong thermodynamic incompatibility with SSEs. Within this oxide subset, the spread across layered compositions (from Co/Mn‐containing NMC to Ni‐rich and Co‐free/Al‐stabilized variants) is secondary compared with the overall separation from polyanionic cathodes, whereas high‐voltage oxide chemistries tend to populate the most reactive region of the map. Across the electrolyte axis, differences among thiophosphate frameworks (Li_3_PS_4_, Li_7_P_3_S_11_), LGPS‐type conductors, and argyrodite compositions are present but smaller than the cathode‐driven contrast, suggesting that cathode chemistry is a dominant factor in setting the thermodynamic propensity for interfacial decomposition. Overall, Figure 1a highlights that intrinsically high‐reactivity OC–SSE pairs are prevalent, reinforcing the need for coating strategies that can buffer oxide–sulfide contacts and suppress decomposition driving forces.

Given the sparsity of intrinsically compatible OC–SSE pairs, we next evaluated whether stable Li‐containing compounds can serve as interfacial coatings that reduce the thermodynamic driving force on both sides. Across 809 phase‐stable candidates, the distribution of reactivity is highly skewed toward unfavorable values (Figure 1b). Under a stringent low‐reactivity criterion of *E_reaction_* ≥ ‐50 meV/atom (i.e., weak driving force in magnitude), only 53 candidates satisfy the threshold, indicating that thermodynamically promising coatings occupy a small fraction of the available stable composition space. The individual coating–OC and coating–SSE reaction energies for the top 20 candidates are provided in Figure S1. This pronounced sparsity motivates a data‐driven strategy that learns compositional signatures of low–*E_reaction_* behavior and extrapolates beyond database entries to systematically expand and screen coating compositions.

### 2.2 Reactivity‐Profile Clustering and Compositional Fingerprinting

**FIGURE 2:**
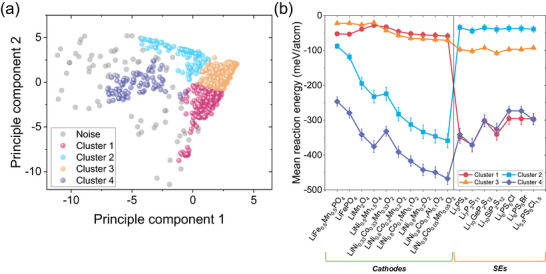
Clustering analysis and stability evaluation. (a) Two‐dimensional PCA projection of the reactivity‐profile space. Gray points indicate noise identified via HDBSCAN, while colored points denote clusters determined by k‐medoids. (b) Mean mutual reaction energies for each cluster against 10 oxide cathodes and 7 sulfide SEs. Error bars represent the 95% confidence interval.

For the 809 phase‐stable Li‐containing compounds, we applied a data‐driven unsupervised learning workflow to classify coating candidates by their interfacial reactivity profiles (Figure 2). Each candidate was represented in the full *E_reaction_* descriptor space constructed from its *E_reaction_* values against the 10 oxide cathodes and 7 SSEs, such that materials with similar cathode and electrolyte reactivity patterns were grouped together. Low‐density samples were first identified as noise by HDBSCAN and excluded, after which k‐medoids clustering (k = 4) separated the remaining candidates into four distinct groups (Clusters 1 to 4, Figure 2a). This medoid‐based partitioning is well‐suited to reaction‐energy profiles, which can exhibit heavy‐tailed distributions and occasional extreme values/outliers that can bias centroid‐based clustering [40, 41]. By selecting cluster representatives from the observed candidates (medoids), the resulting groups remain anchored to chemically realizable coatings and become less sensitive to outliers in the high‐dimensional *E_reaction_* space. To support this choice, we benchmarked multiple unsupervised clustering methods (Ward, spectral, GMM, Leiden, and k‐medoids) and selected k‐medoids based on the joint criteria of high silhouette score and controlled cluster‐size imbalance quantified by the relative standard deviation (RSD; *σ/µ*) of cluster populations (Figure S2). Principal component analysis (PCA) was used only to visualize these high‐dimensional relationships in two dimensions, revealing well‐separated regions that correspond to different interfacial‐stability regimes.

The resulting clusters exhibit clearly differentiated *E_reaction_* trends across both cathodes and SSEs (Figure 2b, mean values with 95% confidence intervals). Clusters 1 and 3 show consistently less negative *E_reaction_* across oxide cathodes, indicating comparatively weaker thermodynamic driving forces for cathode side decomposition, whereas Clusters 2 and 4 display more cathode–sensitive behavior with substantially more negative *E_reaction_* for specific cathodes, reflecting stronger and more variable reactivity patterns. In contrast, the electrolyte side separates the clusters in a different way: Cluster 2 maintains the least negative *E_reaction_* across all SSEs, consistent with a coating family that is broadly compatible with sulfide chemistries, while Cluster 1 shows a pronounced drop to more negative values upon moving from the cathode set to the SSE set, indicating a trade‐off where candidates that are relatively benign toward oxides can still be reactive toward sulfides. Cluster 4 remains the most reactive regime overall, with persistently negative *E_reaction_* against both material sets. Representative compositions illustrating each cluster, together with their side‐specific mean reaction energies and main chemical families, are listed in Table S1 to provide concrete examples of the grouped reactivity behavior. Together, these trends demonstrate that the clustering captures not only average stability but also the coupled and asymmetric nature of coating compatibility with cathodes versus electrolytes, motivating the use of cluster level chemical fingerprints for interpretable design rules and subsequent virtual composition search.

**FIGURE 3:**
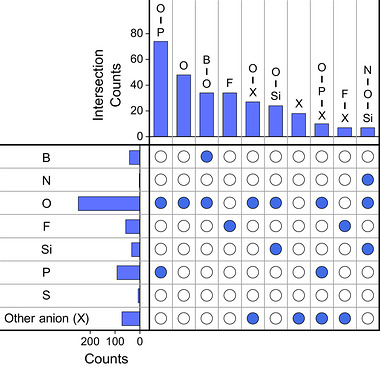
Anion‐composition fingerprints of Cluster 3 shown as an UpSet plot. The left bars report the number of compounds containing each individual anion, and the top bars give the intersection counts for the ten most frequent anion combinations. The dot matrix indicates the anions included in each combination (filled dots), with open dots denoting absence.

Cluster‐level fingerprinting (Figure S3) reveals distinct anion chemistries across the four groups: Cluster 1 is strongly oxygen‐rich (O presence ≈ 0.96) with noticeable O─Sb and O─Se co‐occurrence, Cluster 2 is a mixed‐anion group with substantial S presence (≈ 0.46) and non‐negligible N and P contributions, including pairings such as P─S and P─Se, and Cluster 4 is marked by a pronounced nitrogen signature (N presence ≈ 0.76) accompanied by N–Si co‐occurrence. These fingerprints are consistent with the representative compositions in Table S1, where Cluster 1 includes O‐rich oxide and tungstate families, Cluster 2 includes mixed‐anion oxynitride and cyanamide families, and Cluster 4 includes non‐oxide antimonide and nitride‐related families. In contrast, Cluster 3 (310 compounds), which exhibits the weakest interfacial driving forces against both cathodes and SSEs, forms an oxygen‐centered family enriched with oxygen–polyanion families, highlighted by strong O─P co‐occurrence (≈ 0.30) and appreciable B─O and O─Si signals (Figure S3). Representative Cluster 3 compositions include oxide and oxide‐polyanion compositions with phosphate/pyrophosphate, Zr‐oxide, and silicate families, consistent with this oxygen‐centered fingerprint (Table S1). The anion‐composition UpSet plot for Cluster 3 (Figure 3) further shows that, beyond O‐only compositions, the most frequent anion intersections include A–O combinations where *A = B, P, or Si*, indicating systematic incorporation of polyanion‐forming elements within an oxygen framework. This oxygen–polyanion signature provides an interpretable compositional envelope for subsequent candidate‐set expansion and ML‐guided screening in the Li─M─O and Li─M─A─O search spaces.

### 2.3 Supervised Surrogate Modeling of Mean Interfacial Reaction Energy

Using the composition‐derived physicochemical descriptors, we next trained supervised regressors to predict the mean interfacial reaction energy averaged over all cathode and SSE pairings. Model comparison in the accuracy–stability map (Figure S4) identified XGBoost (XGB) as the best performer (lowest *MAE_CV_*) and histogram‐based gradient boosting (HGBR) as the most stable choice among models with below‐median *MAE_CV_* (smallest generalization gap, Δ*MAE*  =  *MAE_CV_* − *MAE_train_*). This separation is consistent with the inductive biases of the two boosting frameworks: XGB can more aggressively exploit high‐order, nonlinear interactions and threshold‐like dependencies among composition descriptors [42, 43], which often improves average error metrics, whereas HGBR's feature binning and constrained split search act as an implicit regularizer that reduces sensitivity to resampling and yields more reproducible generalization [44, 45].

**FIGURE 4:**
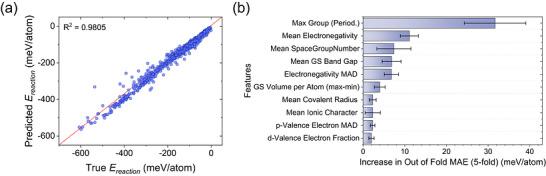
Predictive performance and feature‐importance analysis. (a) In‐sample parity plot for the XGB+HGBR ensemble retrained on the full labeled dataset (consistency check), comparing predicted and reference mutual reaction energies; the dashed line indicates perfect agreement. (b) Top 10 features ranked by out‐of‐fold permutation importance, quantified as the increase in MAE upon feature permutation across five‐fold cross‐validation (error bars show fold‐to‐fold variability).

As a result, combining XGB (accuracy) and HGBR (stability) provides a practical balance for prediction‐guided screening, where both low prediction error and robust candidate ordering based on predicted values are important under limited labeled data. We therefore combined these two complementary predictors into a simple weighted ensemble, using 5‐fold out‐of‐fold predictions to optimize the mixing weight and obtaining weights of 0.44 (XGB) and 0.56 (HGBR). The final ensemble was retrained on the full labeled dataset for screening, and it reproduces the reference values well in the corresponding in‐sample parity plot (Figure 4a), which serves as a consistency check of the learned composition‐to‐reactivity mapping (cross‐validation and held‐out test performance are summarized in Figure S4).

Feature‐importance analysis based on out‐of‐fold permutation (Figure 4b) indicates that predictive performance is primarily supported by composition‐derived descriptors associated with periodic group and valence electron statistics, bonding and ionicity, and size and packing. Representative features include the highest periodic group among constituent elements, the dispersion of p valence electron counts, the fraction of d valence electrons, the mean and dispersion of electronegativity, average ionic character, elemental band gap statistics, the spread in elemental ground state volume per atom, and the mean covalent radius. These descriptors capture broad composition‐level distinctions in anion chemistry, bonding character, redox flexibility, and constituent size mismatch, which also differentiate the and cluster‐defined families. Because permutation importance quantifies predictive utility within the trained model rather than causality, these descriptors should be interpreted as composition level proxies rather than deterministic design rules, independent stability scores, or direct crystallographic and interface structural descriptors. A dedicated causal attribution of individual descriptors remains beyond the scope of this work.

### 2.4 ML‐Guided Candidate‐Set Expansion for Low‐Reactivity Coatings

**FIGURE 5:**
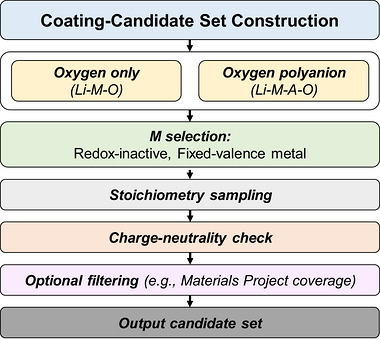
Schematic workflow for constructing an expanded set of coating‐composition candidates guided by cluster‐derived compositional signatures.

Building on the cluster‐derived compositional signatures, we constructed an ML‐guided candidate‐set expansion workflow (Figure 5) that systematically enumerates charge‐balanced Li─M─O and Li─M─A─O compositions within the oxygen‐centered, polyanion‐compatible design envelope identified by unsupervised analysis. In this workflow, the Li─M─O and Li─M─A─O composition spaces were derived from the low‐reactivity cluster signatures. In particular, the Li─M─A─O space was motivated by the enrichment of oxygen‐polyanion motifs in the low‐reactivity cluster, with B, P, and Si selected as representative polyanion‐forming elements. The M‐element pool, stoichiometric sampling rules, and charge‐neutrality requirement based on nominal oxidation states were then introduced as empirical chemical constraints to generate plausible coating compositions within this envelope. These rules define the chemically controlled candidate space rather than assigning independent stability scores.

**FIGURE 6:**
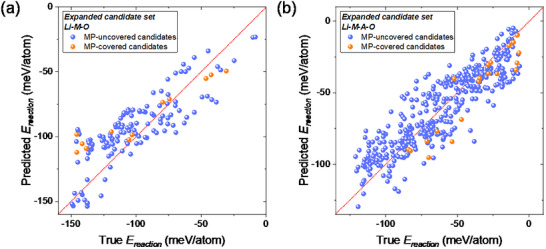
Prediction performance for Li‐based coating candidates in the expanded composition set: (a) Li─M─O and (b) Li─M─A─O. Blue markers denote candidates not reported in the Materials Project database, while orange markers indicate candidates covered by the Materials Project. The dashed line corresponds to the parity line (*y = x*).

The trained ensemble model was then used to predict the mean interfacial reaction energy for each composition in the expanded candidate set. Candidate prioritization was subsequently performed based on these predicted values, with less negative predicted reaction energies indicating weaker thermodynamic driving forces for interfacial decomposition. For down‐selection, we additionally computed two side‐specific mean reaction energies, ⟨*E_reaction_* ⟩_OC_ and ⟨*E_reaction_* ⟩_SSE_, by averaging the reaction energies over the cathode set and the SSE set separately, and prioritized candidates that remain low‐reactivity against both sets to avoid compositions that are favorable only on one side. Figure 6 evaluates the resulting predictions by comparing the predicted versus thermodynamically evaluated interfacial reaction energies for the expanded candidates, separately for (a) Li─M─O and (b) Li─M─A─O, with the parity line indicating ideal agreement. For compositions not reported in the Materials Project (blue markers), direct target phase energies are not available. Therefore, we constructed precursor‐based proxy labels by decomposing each target composition into a set of Materials Project stable precursor phases on the convex hull and evaluating the corresponding phase diagram reaction energies for the resulting assemblage. These proxy labels should be interpreted as composition level thermodynamic consistency checks rather than exact reaction energies of synthesized target phases. Within this proxy evaluation framework, Materials Project unreported candidates show an overall parity trend similar to Materials Project covered compositions, indicating that the supervised model provides ranking level consistency beyond direct database lookup.

To further examine whether the expanded composition set represents uncontrolled extrapolation, we performed an applicability domain analysis in the same standardized composition descriptor space used for supervised learning (Figure S5). The applicability domain distance was defined as the average Euclidean distance to the five nearest reference compounds after scaling descriptors using only the 809 reference compounds. Using the 90, 95, and 99th percentiles of the reference leave one out distance distribution as thresholds, 94.6% of the expanded compositions were located within the core reference domain and 97.9% were located within the boundary reference domain, with no composition entering the extreme extrapolative region. The small number of compositions above the 95th percentile correspond to sparsely covered regions of the reference descriptor space, including some lanthanide‐containing compositions within the predefined expansion space. These results provide a quantitative basis for distinguishing database expansion from descriptor space extrapolation. Accordingly, ML predictions for the expanded composition set are interpreted as ranking and prioritization scores within this chemically constrained space, rather than as precise quantitative estimates of absolute reaction energies. The ML framework should be viewed as a thermodynamically grounded first‐pass filter for ranking and prioritizing coating compositions for follow‐up validation, rather than a definitive predictor of precise interfacial reaction pathways or practical coating performance.

**FIGURE 7:**
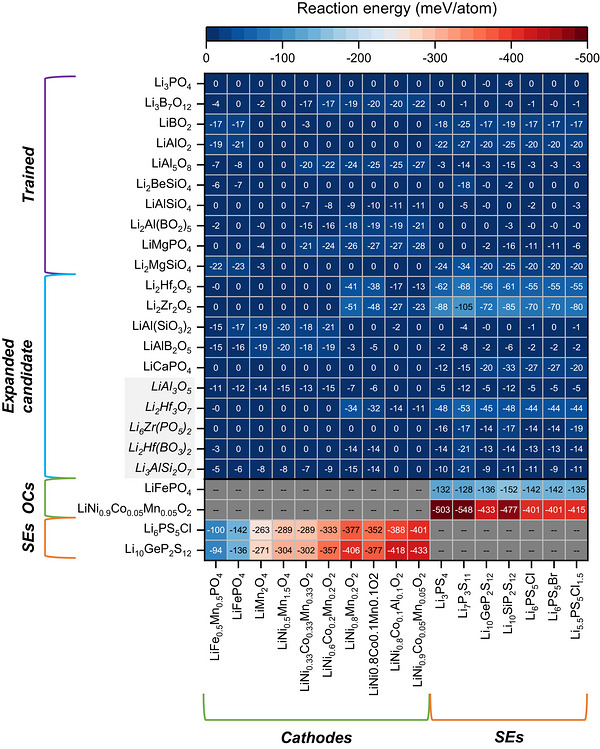
Heatmap of mutual reaction energies between lithium solid electrolytes (SSEs) and cathodes evaluated with coating compositions from the training set and the expanded candidate set. Compositions shown in *gray italics* are candidates not reported in the Materials Project database. The bottom two rows provide the direct cathode–SSE mutual reaction energies for reference.

Figure 7 provides a consolidated heatmap that compares interfacial reaction energies for coatings drawn from both the oxide‐rich training set and the cluster‐guided expanded candidate set constructed under the Li─M─O and Li─M─A─O composition rules. The expanded candidates shown here were selected using the joint *⟨E_reaction_⟩_OC_* and *⟨E_reaction_⟩_SSE_* criteria to ensure balanced compatibility with both oxide cathodes and sulfide electrolytes. In the heatmap, the top ten rows correspond to selected training‐set coatings, followed by five expanded candidates covered by the Materials Project, five expanded candidates not covered by the Materials Project (gray italics), two cathodes, and two SSEs; the cathode and SSE rows are included as references to directly contrast coated versus uncoated oxide–sulfide contacts. The bottom reference rows highlight that direct cathode–SSE interfaces are strongly reactive, with reaction energies averaging ≈ −332 meV/atom, underscoring the large thermodynamic driving forces for interfacial decomposition without coatings. In contrast, the coating layers substantially reduce reaction driving forces on both sides: across the coating compositions shown in Figure 7, the mean coating–cathode reaction energy is ≈ −8 meV/atom (range −51 to 0 meV/atom) and the mean coating–SSE reaction energy is ≈ −17 meV/atom (range −105 to 0 meV/atom). These reductions demonstrate that the candidate‐set expansion and ML‐guided screening strategy identifies coatings that can simultaneously buffer both interfaces, transforming highly reactive cathode–SSE pairings into substantially less reactive coating‐mediated contacts.

Our study evaluates interfacial compatibility using reaction energies calculated from bulk thermodynamic data. While this approach provides a robust and scalable metric for high‐throughput screening, it does not by itself capture several factors that can critically influence practical coating behavior, including reaction kinetics, interfacial microstructure, coating thickness and conformity, adhesion, space‐charge‐layer formation, strain and volume‐change effects, and Li‐ion transport across the coating layer. By subtracting the self‐decomposition energy of each solid phase, our metric strictly quantifies the thermodynamic driving force arising from the contact between two phases. Therefore, these values serve as comparative indices of stability rather than precise forecasts of reaction products, interfacial morphologies, or transport behavior formed under non‐equilibrium processing or battery operating conditions.

Despite the absence of kinetic parameters, thermodynamic screening remains a proven primary filter for identifying stable solid‐state battery interfaces. It highlights cases where protective coatings are essential to prevent highly exergonic reactions that lead to impedance rise. Building on this foundation, we integrate ML to broaden the scope of materials discovery. By utilizing cluster‐based compositional features, our ML model defines a clear search space, allowing for the rapid and thermodynamically consistent prediction of novel compositions beyond existing databases. Future work will need to bridge the gap between these thermodynamic predictions and practical application by factoring in finite‐temperature stability, metastability, reaction kinetics, space‐charge effects, strain/volume changes, interfacial transport, and ion‐transport properties.

Although Li‐ion conductivity was not explicitly evaluated in the present framework, the present screening uses thermodynamic compatibility to identify coating compositions that can suppress interfacial decomposition. Modest bulk Li‐ion conductivity does not necessarily preclude the use of a material as a thin conformal coating, for which the transport penalty strongly depends on coating thickness and interfacial resistance. Representative coating materials such as Li_3_PO_4_ and LiNbO_3_ have often been investigated as interfacial coating layers despite their modest bulk Li‐ion conductivities, particularly when used as sufficiently thin layers [46]. Consistent with this thin coating perspective, reported Li‐ion conductivity values for related phosphate, niobate, silicate, borate, and lithium metal oxide families span a broad, chemistry‐dependent range (Table S2). These literature values provide chemical family level context for transport relevance, but they are not assigned as conductivities of the individual expanded compositions. Accordingly, Li‐ion transport should be considered in subsequent validation when assessing the practical applicability of the thermodynamically prioritized compositions.

From a translational perspective, the top‐ranked compositions identified here should be regarded as thermodynamically prioritized targets for follow‐up validation rather than final synthesis‐ready coating materials. Depending on the target chemistry, they could be examined through precursor‐based synthesis, wet‐chemical coating, or thin‐film deposition approaches, either as coating phases or as interfacial layers on representative oxide cathode particles. Subsequent validation should combine phase and interfacial characterization to examine phase formation, coating morphology, and interfacial reaction products. Electrochemical measurements could then be used as follow‐up assessments to evaluate the practical consequences of suppressed interfacial decomposition under cell‐relevant conditions. Additional computational checks, such as electrochemical stability‐window and Li‐ion migration analyses, could further support candidate down‐selection before experimental testing.

## 3 Conclusions

In conclusion, we developed an ML‐guided workflow that transforms database‐limited thermodynamic screening into an interpretable candidate‐set expansion strategy for stabilizing oxide–sulfide interfaces in all‐solid‐state Li‐ion batteries. Phase‐diagram‐based interfacial reaction energies for 10 oxide cathodes and 7 sulfide solid electrolytes show that intrinsically low‐reactivity OC–SSE pairings are rare, with direct contacts exhibiting strong driving forces (average ≈ −332 meV/atom), motivating the need for coatings. Starting from 809 phase‐stable Li‐containing compounds, unsupervised clustering of reaction‐energy profiles separated candidates into distinct reactivity groups; mapping these groups in composition space revealed consistent compositional signatures and identified an oxide‐compatible, polyanion‐tolerant chemical regime as broadly low‐reactivity. Using composition‐derived physicochemical descriptors, we trained a supervised ensemble predictor (XGB + HGBR) to estimate the overall mean interfacial reaction energy over OC–SSE pairings and applied it to screen systematically enumerated Li─M─O and Li─M─A─O compositions, including candidates beyond Materials Project coverage. Importantly, the expansion step was driven by the cluster‐derived signatures, while the surrogate model served to prioritize candidates within this precursor‐consistent thermodynamic evaluation framework. Across representative coatings from both the training set and the expanded candidates, the interfacial driving forces were substantially reduced on both sides, with mean coating–cathode reaction energies of about ‐8 meV/atom (range −51 to 0) and mean coating–SSE reaction energies of about −17 meV/atom (range −105 to 0).

## 4 Methods

First‐principles‐based phase structures and total energies were obtained from the Materials Project database through the Python Materials Genomics (Pymatgen) library [47, 48, 49]. An initial pool of Li‐containing compounds was collected using composition‐based filters. To focus on phases that are thermodynamically stable against decomposition, candidates were screened by the energy above the convex hull (*E*_hull_). Only entries with *E*_hull_ = 0 meV/atom were retained as coating candidates, yielding 809 compounds. Although finite‐temperature effects and metastability can be experimentally relevant, restricting to *E*_hull_ = 0 provides a consistent baseline for learning and systematic comparison.

Interfacial chemical compatibility is assessed using a corrected phase‐diagram driving force, denoted here as *E_reaction_* [50, 51, 52, 53]. For a contact pair *A* and *B*, we parameterize the overall composition by the molar fraction *x* (0< *x* < 1). The energy of the pseudo‐binary mixture is defined as:

(1) $$E_{bin}\;\left(x\right)=xE_{A}+\left(1-x\right)E_{B}$$

At the same overall composition *C_bin_* (*x*)  =  *xC_A_* + (1 − *x*)*C_B_*, the phase‐equilibrated energy *E_eq_* (*C_bin_* (*x*)) is obtained as the minimum energy over all competing phase assemblages from the multicomponent phase diagram. The uncorrected decomposition driving force is

(2) $$\Delta E_{D}\;\left(x\right)=E_{eq}\;\left(C_{bin}\left(x\right)\right)-E_{bin}\left(x\right)$$

To isolate the additional driving force associated with bringing two solids into contact, we subtract the intrinsic decomposition energies of the individual compounds, Δ*E_D_* (*A*) and Δ*E_D_* (*B*), evaluated within the same phase‐diagram reference, and define

(3) $$E_{reaction}\;\left(x\right)=\Delta E_{D}\left(x\right)-x\Delta E_{D}\left(A\right)-\left(1-x\right)\Delta E_{D}\left(B\right)$$

With this sign convention, a more negative *E_reaction_* indicates a stronger thermodynamic driving force for interfacial decomposition beyond intrinsic instabilities. We evaluate *E_reaction_(x)* over 0< *x* < 1, report the minimum value (most negative) as the representative interfacial driving force, and normalize it per atom (meV/atom). Accordingly, *E_reaction_* ≈ 0 meV/atom indicates a negligible additional driving force for cross‐reaction. This contact‐reaction metric is distinct from the intrinsic electrochemical stability window of an isolated electrolyte or coating phase. Direct oxide cathode–sulfide solid electrolyte reaction energies are used to assess the thermodynamic need for coatings, whereas coating–cathode and coating–electrolyte reaction energies, together with ⟨*E_reaction_* ⟩_OC_ and ⟨*E_reaction_* ⟩_SSE_, are used to prioritize candidates compatible with both contacting phases. A threshold of *E_reaction_* ≥ −50 meV/atom is used as a practical low‐reactivity criterion.

The cathode set comprises ten Li intercalation cathodes selected to span widely used positive‐electrode structure families and redox chemistries [54, 55, 56, 57, 58, 59]: olivine phosphates (LiFePO_4_ and LiFe_0.5_Mn_0.5_PO_4_), spinel oxides (LiMn_2_O_4_ and the high‐voltage spinel LiNi_0.5_Mn_1.5_O_4_), and layered $R\bar{3}m$ oxides. The layered subset includes Co/Mn‐containing NMC compositions (LiNi_0.33_Co_0.33_Mn_0.33_O2 and LiNi_0.6_Co_0.2_Mn_0.2_O_2_) and Ni‐rich variants (LiNi_0.8_Co_0.1_Mn_0.1_O_2_ and LiNi_0.9_Co_0.05_Mn_0.05_O_2_), as well as Co‐free and Al‐stabilized analogs (LiNi_0.8_Mn_0.2_O_2_ and LiNi_0.8_Co_0.1_Al_0.1_O_2_), capturing systematic changes in Ni content and transition‐metal substitution that modulate redox chemistry and oxygen activity, which can influence interfacial reactivity. The SSE set includes seven sulfide‐based solid electrolytes covering major thiophosphate frameworks and halogen substitution effects [60, 61, 62, 63, 64, 65]: Li_3_PS_4_, thio‐LISICON‐related Li_7_P_3_S_11_, LGPS‐type superionic conductors (Li_10_GeP_2_S_12_ and Li_10_SiP_2_S_12_), and argyrodite phases with varying halide content (Li_6_PS_5_Cl, Li_6_PS_5_Br, and Li_5.5_PS_5_Cl_1.5_). Together, these choices represent practical oxide‐cathode and sulfide‐electrolyte pairings while sampling key cathode and electrolyte chemistries that govern the thermodynamic driving forces for interfacial decomposition.

To enable screening and prediction for hypothetical compositions whose crystal structures are unknown, we use composition‐derived descriptors rather than structure‐dependent features [66, 67]. Feature vectors were constructed with Matminer featurizers [66], including stoichiometric descriptors, the MAGPIE elemental property preset [67], which aggregates elemental attributes (e.g., electronegativity, covalent/atomic radii, and valence‐electron counts) into composition‐level statistics (mean, range, and variance weighted by atomic fractions). Additional featurizers were used to capture valence‐orbital character and ionic‐property proxies relevant to charge compensation and anion chemistry.

In addition, we considered composition‐level quantities related to chemical motifs and plausibility constraints relevant to candidate screening, including the number of elements, ideal mixing entropy, anion atomic fractions, polyanion‐element fractions (e.g., B/P/Si), transition‐metal‐related indicators, and charge‐balance consistency estimated from nominal oxidation states. These quantities were used to describe or constrain the search space, but were not treated as independent stability scores or deterministic design rules. In particular, the expanded Li─M─O and Li─M─A─O candidate spaces were constructed from the cluster‐derived oxygen‐centered and oxygen‐polyanion signatures, while the M‐element pool, stoichiometric sampling rules, and charge‐neutrality requirement were used as empirical constraints to generate chemically plausible compositions.

These descriptors were used for supervised prediction of the mean interfacial reaction energy, and the resulting predicted values were subsequently used for candidate prioritization and down‐selection in the expanded composition set. Because the descriptors used for supervised prediction are derived from composition or aggregated elemental properties, they should be interpreted as composition‐level proxies rather than direct crystallographic or interface‐structural descriptors of the target compositions.

Unsupervised learning was used to group coating candidates into chemically interpretable families and relate these families to trends in interfacial reaction energy. For clustering, each candidate was represented by its reactivity profile, i.e., the vector of interfacial reaction energy values computed against the cathode and SSE sets (after appropriate normalization), so that candidates were grouped by similarity in interfacial thermodynamic behavior rather than by crystal‐structure information. We first applied HDBSCAN [68], a density‐based clustering algorithm, to the reaction‐energy profile vectors in order to identify sparse, low‐density outliers. Points labeled as noise were excluded from subsequent partitioning so that downstream clustering captured the dominant, reproducible patterns in interfacial reactivity. We then evaluated multiple clustering approaches on the remaining candidates, including Ward hierarchical clustering [69], spectral clustering [70], Gaussian mixture models [71, 72], Leiden graph clustering [73], and k‐medoids [40]. The final clustering approach was selected based on (i) the silhouette score as an internal measure of cluster separation and (ii) cluster‐size imbalance quantified by the relative standard deviation (RSD; *σ/µ*) of cluster populations. Based on these criteria, we adopted k‐medoids with *k = 4*, which achieved strong separation without severe size imbalance (see Figure S2). To facilitate chemical interpretation and downstream design, we defined a compositional fingerprint for each cluster using element occurrence statistics and anion‐pair co‐occurrence statistics, which subsequently served as the basis for candidate‐set expansion in the targeted Li─M─O and Li─M─A─O design spaces.

Supervised regression models were trained to predict the overall mean interfacial reaction energy for each coating composition, defined as the mutual reaction energy averaged over all oxide cathodes and sulfide solid electrolytes (SSEs). We compared seven regressors: Extra Trees (ET) [74], Random Forest (RF) [75], histogram‐based gradient boosting regressor (HGBR) [44, 45, 76], XGBoost (XGB) [42], LightGBM (LGBM) [76], CatBoost (CAT) [77, 78], and ElasticNet (ENet) [79]. The dataset was split once into training (80%) and held‐out test (20%) sets; the test set was used only for final evaluation. After this final evaluation, we retrained the selected models on the full labeled dataset to build the screening predictor used for candidate‐set expansion. Hyperparameters were optimized on the training set using 5‐fold cross‐validation (CV) with an identical search budget (54 configurations) for each model. Model selection used a penalized objective that balances accuracy and generalization,

(4) $$Penalized\;Score=MAE_{CV}+\max\left(0,\;\left(MAE_{CV}-MAE_{train}\right)\right)$$

where the second term discourages overfitting when validation error exceeds training error. We further identified two complementary leaders from cross‐validation results: a performance leader minimizing *MAE_CV_*, and a stability leader minimizing the generalization gap among models with *MAE_CV_* no worse than the median. The final predictor was an OOF‐optimized weighted ensemble of XGBoost and HGBR, where the mixing weight *w* was selected by MAE‐minimizing grid search using 5‐fold out‐of‐fold predictions, and the screening predictor was obtained by retraining both models on the full labeled dataset and computing. *Y_ensemble_* =  *wY_XGB_* + (1 − *w*)*Y_HGBR_*. In our benchmark, XGB achieved the lowest *MAE_CV_*, whereas HGBR showed the smallest gap among competitive models (see Figure S4); thus, the final ensemble combines XGB and HGBR to balance accuracy and robustness when screening the expanded candidate sets derived from cluster signatures. In addition to the overall mean target used for regression, we computed two side‐specific mean reaction energies, *⟨E_reaction_⟩_OC_* and *⟨E_reaction_⟩_SSE_*, by averaging the reaction energies over the cathode set and the SSE set separately, and used them as joint down‐selection criteria for the expanded candidate sets to ensure balanced compatibility with both contacting phases. Feature contributions were quantified using permutation importance computed in a 5‐fold out‐of‐fold framework. For each fold, we measured the increase in MAE after permuting each feature on the validation split (Δ*MAE*), repeating permutations 10 times to reduce variance. The top features are reported with fold‐wise variability (standard deviation).

Virtual coating compositions were generated to systematically expand the candidate set beyond existing database entries. Two target spaces were considered: ternary oxygen‐only compositions (Li─M─O) and quaternary oxygen–polyanion compositions (Li─M─A─O), where *M* denotes a redox‐inactive or fixed‐valence metal and *A* denotes a polyanion‐forming element (e.g., B, P, or Si). The *M* element pool (Mg, Ca, Al, Zn, Ga, Zr, Hf, Sc, Y, La, Gd, Dy, Ho, Er, Lu) was designed to favor oxide‐based coatings by prioritizing cations with limited oxidation‐state variability and strong oxide‐forming tendencies, thereby reducing redox‐driven interfacial reaction pathways and maintaining a consistent oxide‐chemistry regime for thermodynamic screening. Mg, Ca, and Zn were retained as representative fixed‐valence (2+) cations that enlarge the charge‐balanced design space while avoiding variable‐valence transition‐metal chemistry; moreover, their oxide chemistry remains closer to the targeted inert‐coating regime than that of highly polarizable, very large alkaline‐earth cations. The pool spans small, strongly bonding cations (e.g., Al/Ga/Zr/Hf) and larger trivalent stabilizers (e.g., Y and lanthanides) to broaden compositional coverage within this design envelope. For each target space, stoichiometries were sampled randomly under predefined bounds on element fractions and total atom counts, and charge neutrality was enforced using nominal oxidation‐state assignments. Candidates failing the charge‐balance constraint were discarded. An optional filtering step was applied depending on the analysis goal, for example, excluding compositions already present in the Materials Project to focus screening on the expanded candidate set. For MP‐unreported candidates, we evaluate thermodynamic values as precursor‐based proxy labels by decomposing the target composition into a convex‐hull assemblage of MP‐stable phases and computing phase‐diagram reaction energies for that assemblage.

## Conflicts of Interest

The authors declare no conflict of interest.

## Supporting information




**Supporting File**: advs76305‐sup‐0001‐SuppMat.pdf.

## Data Availability

The data that supports the findings of this study are available in the supplementary material of this article.
