# Supplementary material for: Machine‐Learning Framework for Designing Stable Interfaces in All‐Solid‐State Lithium‐Ion Batteries
Source: Adv Sci (Weinh). 2026 Jun 26:e76305. Online ahead of print. doi: 10.1002/advs.76305 (PMC13336918; doi:10.1002/advs.76305)
Supplement: Supplementary file 1 — Supporting File: advs76305‐sup‐0001‐SuppMat.pdf. [file ADVS-9999-e76305-s001.pdf]

Supporting Information

Machine-Learning Framework for Designing Stable Interfaces in All-Solid-State Lithium-Ion Batteries

Sehyeok Park, [a†] Myeongcho Jang, [a,b†] Hun-Gi Jung,[a,c,d] Kyung Yoon Chung,[a,c] Seungho Yu,[a,c,*]

[a]Energy Storage Research Center, Korea Institute of Science and Technology, 5, Hwarang-ro 14-gil, Seongbuk-gu, Seoul 02792, Republic of Korea

[b]School of Mechanical Engineering, Korea University, 145 Anam-ro, Seongbuk-gu, Seoul 02841, Republic of Korea

[c]Division of Energy & Environment Technology, KIST School, Korea University of Science and Technology, Seoul 02792, Republic of Korea

[d]Department of Energy Science and KIST-SKKU Carbon-Neutral Research Center, Sungkyunkwan University, Suwon 16419, Republic of Korea

[†]S. P. and M. J. contributed equally to this work.

[*]Corresponding Author

Seungho Yu, E-mail: shyu@kist.re.kr

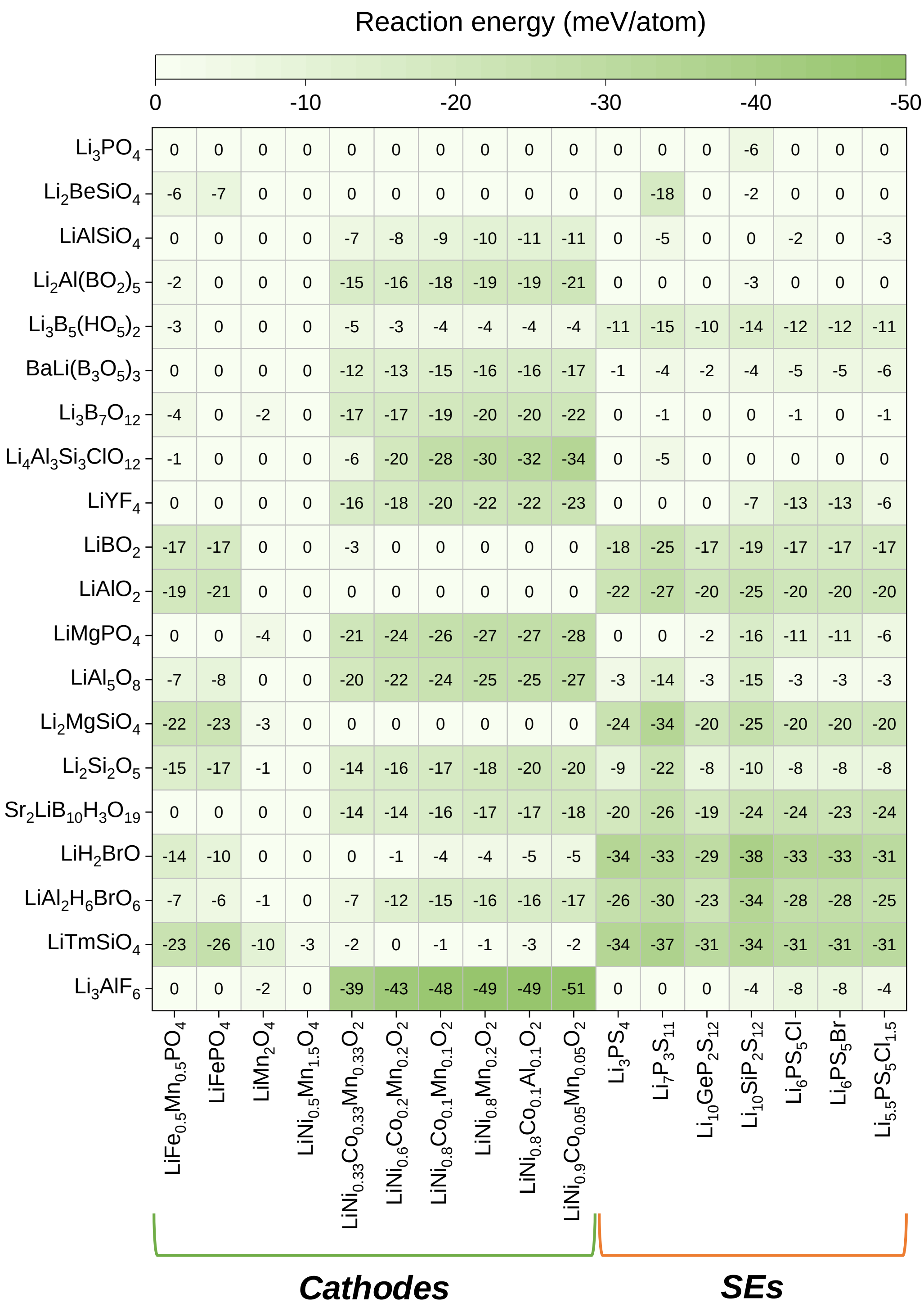

Figure S1. Heatmap of the mutual reaction energies of cathode materials and sulfide SEs against coating material candidates.

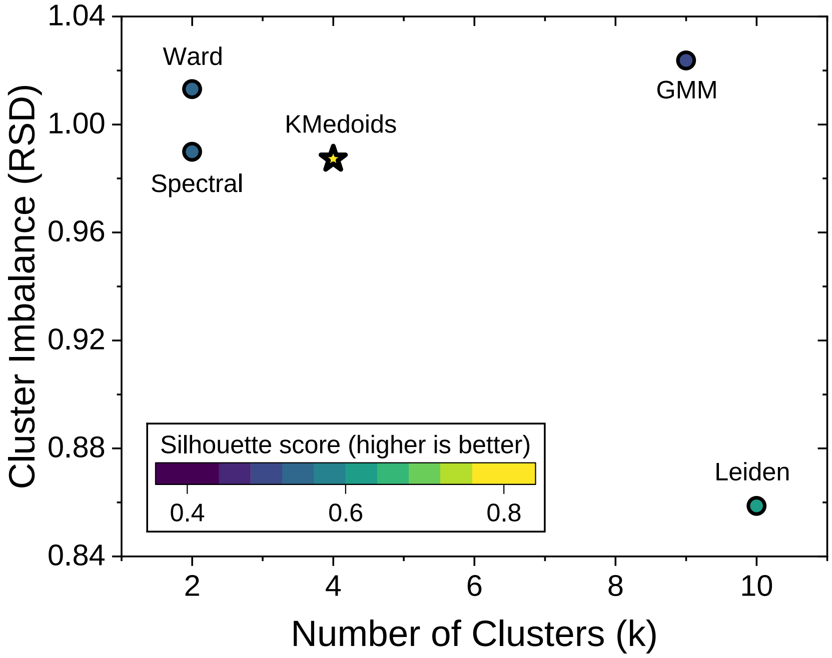

Figure S2. Comparison of unsupervised clustering methods using the number of clusters (k) and cluster-size imbalance quantified by the relative standard deviation (RSD; σ/μ) of cluster populations; marker color indicates the silhouette score (higher is better). The selected setting (k-medoids) is highlighted.

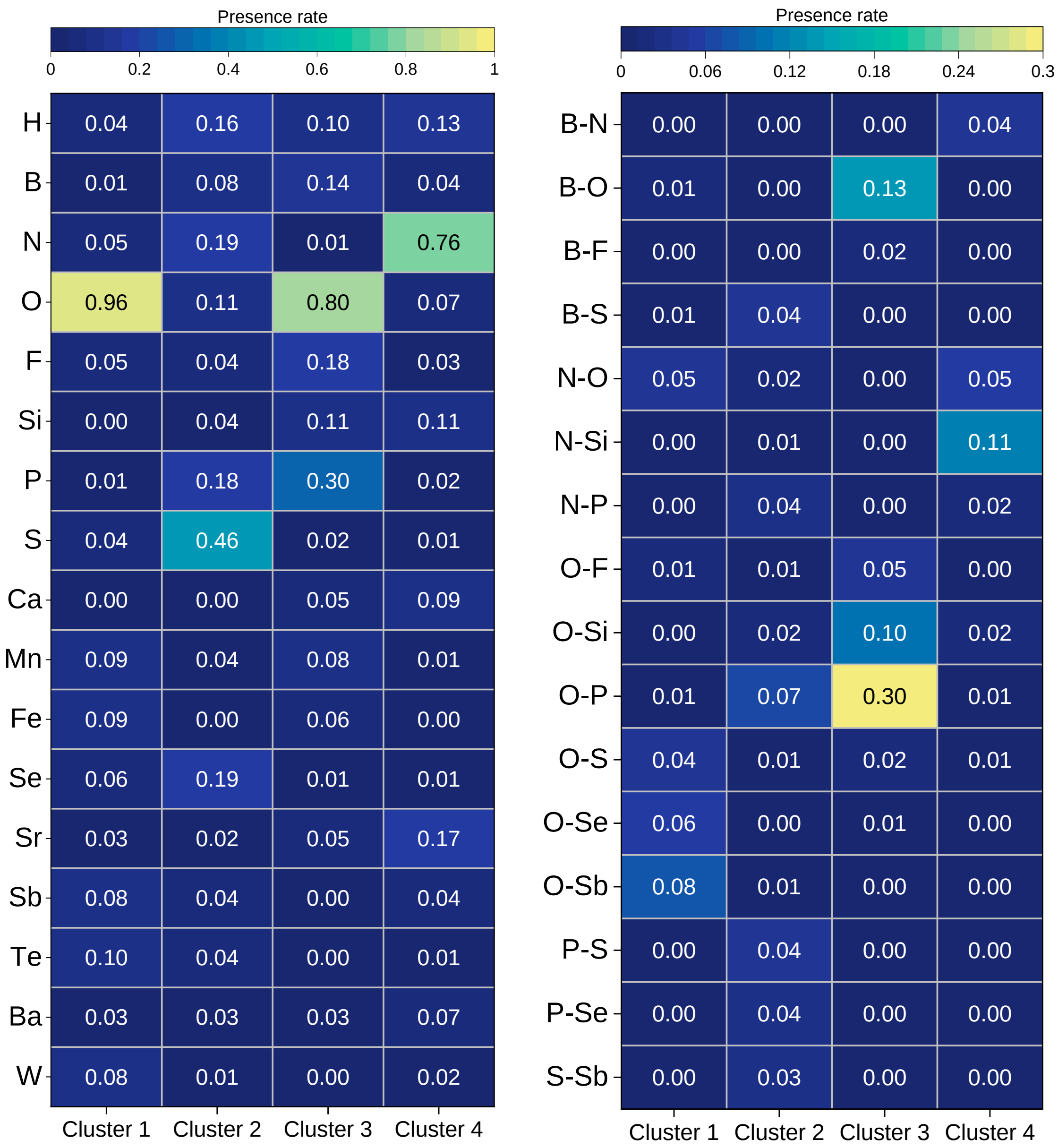

Figure S3. Heatmaps summarizing cluster fingerprints: (left) element presence rate within each cluster and (right) anion-pair co-occurrence rates, where co-occurrence is defined as the fraction of compositions in a cluster that contain both anion species in the given pair.

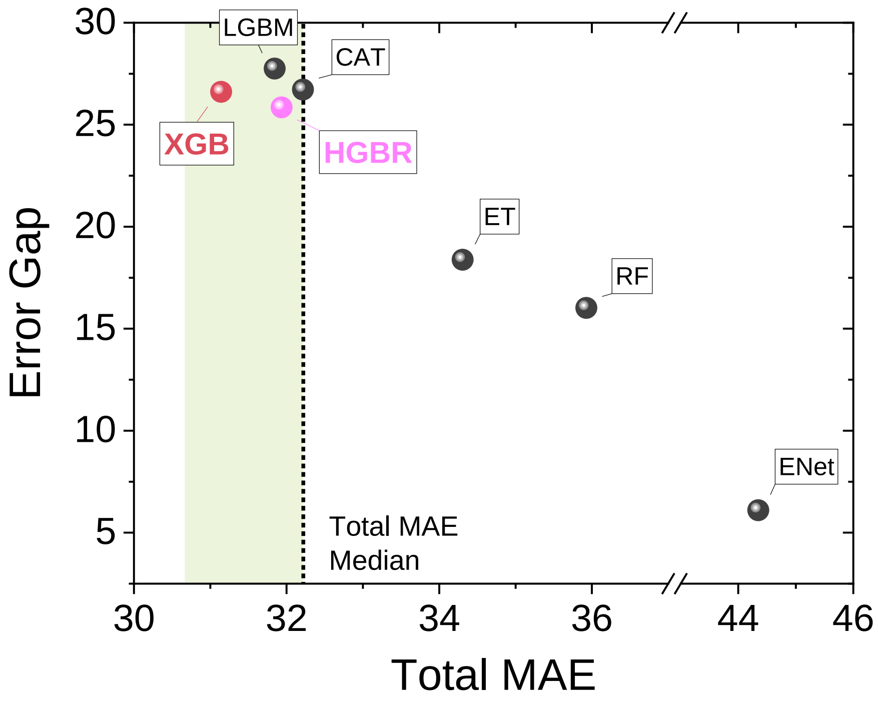

Figure S4. Model selection map showing total MAE (x-axis) versus the generalization gap (y-axis), defined here as the absolute difference between training and test MAE, evaluated using five-fold cross-validation. The shaded region marks models with total MAE below the median across all candidates. The best-performing model (XGB) and the most stable model under the MAE-median constraint (HGBR) are highlighted and combined as the final ensemble predictor.

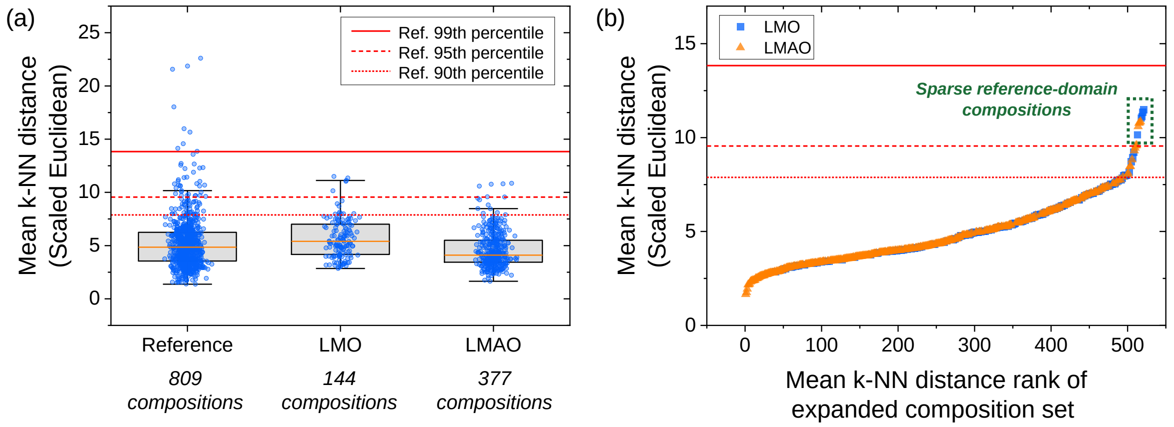

Figure S5. domain analysis of the expanded Li–M–O and Li–M–A–O composition set. (a) Distribution of mean k nearest neighbor distances for the reference set, expanded Li–M–O compositions, and expanded Li–M–A–O compositions. The reference distribution was calculated using leave one out distances, whereas expanded composition distances were calculated relative to the reference set. (b) Sorted mean k nearest neighbor distance spectrum for the expanded composition set.

Note for Figure S5.

Horizontal lines indicate the reference p90, p95, and p99 thresholds, and compositions above the p95 threshold are highlighted as sparse reference domain cases. Applicability domain distances were computed in the z score standardized composition descriptor space used for supervised learning. The 809 reference compounds were used to fit the feature wise mean and standard deviation for standardization, and the same transformation was applied to the expanded composition set. For each composition in the expanded set, the distance was defined as the average Euclidean distance to its five nearest reference compounds. Reference domain thresholds were obtained from the leave one out five nearest neighbor distance distribution of the 809 reference compounds. The 90th, 95th, and 99th percentiles define the core reference domain, boundary reference domain, sparse lower confidence region, and extreme extrapolative region, respectively. Among the 521 compositions in the expanded set, 94.6% were located within the core reference domain and 97.9% were located within the boundary reference domain, with no composition entering the extreme extrapolative region. The upper tail above the p95 threshold corresponds to sparsely covered regions of the reference descriptor space and includes some lanthanide-containing compositions from the predefined expansion space.

Table S1. Representative compositions were selected to reflect both the side-specific reaction-energy behavior and dominant chemical motifs of each cluster. The listed values are the mean reaction energies against the oxide cathode set, $\langle E_{reaction} \rangle_{\mathrm{OC}}$ and the sulfide solid electrolyte set, $\langle E_{reaction} \rangle_{\mathrm{SSE}}$ [unit: meV/atom].

Formula	Cluster	$\langle E_{reaction} \rangle_{\mathrm{OC}}$	$\langle E_{reaction} \rangle_{\mathrm{SSE}}$	Main motif
**Li_5BiO_5**	Cluster 1	-46.9	-330.7	O-rich Bi-oxide
**$LiLa(WO_4)_2$**	Cluster 1	-48.4	-310.9	O-rich tungstate polyanion oxide
**LSiNO**	Cluster 2	-249.3	-39.3	Li–Si oxynitride / mixed (O–N) motif
**$LiAl(CN_2)_2$**	Cluster 2	-262.3	-20.2	cyanamide (C–N) anion framework
**$LiInP_2O_7$**	Cluster 3	-53.2	-114.1	phosphate (O–P) motif
**Li_2ZrO_3**	Cluster 3	-21.5	-111.7	Zr-based oxide / O-rich oxide
**Li_2CaSiO_4**	Cluster 3	-13.4	-97.1	silicate O–Si motif
**LiBaSb**	Cluster 4	-390.4	-303.4	non-oxide antimonide / Zintl-like motif
**Li_3BN_2**	Cluster 4	-355.6	-311.4	nitride / B–N motif

Table S2. Literature based Li-ion transport trends for related chemical families represented in the expanded composition set. Reported values are taken from related coating or electrolyte families and are intended as chemical family level indicators of Li-ion transport relevance. They should not be interpreted as quantitative conductivity assignments to individual expanded compositions.

Chemistry represented in expanded set	Related material or coating family	Reported Li-ion conductivity trend	References
**Li-P-O phosphate family**	e.g., Li_3PO_4	Typically reported in the range of 10^{-6} to 10^{-8} S/cm, depending on structure and processing; often investigated as thin interfacial coating layers	[1]
**Li-Nb-O oxide family**	e.g., $LiNbO_3$	Amorphous-state $LiNbO_3$ is reported around 10^{-5} to 10^{-6} S/cm.; widely investigated as cathode coating/interlayer materials	[2]
**Li-Si-O silicate family**	e.g., Li_2SiO_3, Li_4SiO_4	Reported examples include 10^{-6} to 10^{-8} S/cm level conductivities	[3]
**Li-B-O borate family**	e.g., Li_3BO_3	Reported example is 2×10^{-6} S/cm	[4]
**Li-M-O oxide family**	e.g., $LiAlO_2$, $Li_4Ti_5O_{12}$, Li_2ZrO_3	Broadly chemistry and structure dependent; $LiAlO_2$ (5.6×10^{-8} S/cm), $Li_4Ti_5O_{12}$ (10^{-13} S/cm), Li_2ZrO_3 (10^{-12} S/cm),.	[5,6]

Reference

[1] Y.-Q. Sun, X.-T. Luo, Y.-S. Zhu, X.-J. Liao, C.-J. Li, Li3PO4 electrolyte of high conductivity for all-solid-state lithium battery prepared by plasma spray, J. Eur. Ceram. Soc. 42 (2022) 4239–4247. https://doi.org/10.1016/j.jeurceramsoc.2022.04.010.

[2] W.S.K. Bong, A. Shiota, T. Miwa, Y. Morino, S. Kanada, K. Kawamoto, Effect of thickness and uniformity of LiNbO3-coated layer on LiNi0.5Co0.2Mn0.3O2 cathode material on enhancement of cycle performance of full-cell sulfide-based all-solid-state batteries, J. Power Sources 577 (2023) 233259. https://doi.org/10.1016/j.jpowsour.2023.233259.

[3] A. Nakagawa, N. Kuwata, Y. Matsuda, J. Kawamura, Characterization of Stable Solid Electrolyte Lithium Silicate for Thin Film Lithium Battery, J. Physical Soc. Japan 79 (2010) 98–101. https://doi.org/10.1143/JPSJS.79SA.98.

[4] S. Ohta, S. Komagata, J. Seki, T. Saeki, S. Morishita, T. Asaoka, All-solid-state lithium ion battery using garnet-type oxide and Li3BO3 solid electrolytes fabricated by screen-printing, J. Power Sources 238 (2013) 53–56. https://doi.org/10.1016/j.jpowsour.2013.02.073.

[5] J.S. Park, X. Meng, J.W. Elam, S. Hao, C. Wolverton, C. Kim, J. Cabana, Ultrathin Lithium-Ion Conducting Coatings for Increased Interfacial Stability in High Voltage Lithium-Ion Batteries, Chemistry of Materials 26 (2014) 3128–3134. https://doi.org/10.1021/cm500512n.

[6] M. Wang, Y. Gong, Y. Gu, Y. Chen, L. Chen, H. Shi, Effects of fast lithium-ion conductive coating layer on the nickel rich layered oxide cathode material, Ceram. Int. 45 (2019) 3177–3185. https://doi.org/10.1016/j.ceramint.2018.10.219.
